# Social networks and female reproductive choices in the developing world: a systematized review

**DOI:** 10.1186/1742-4755-11-85

**Published:** 2014-12-10

**Authors:** Samantha MP Lowe, Spencer Moore

**Affiliations:** School of Kinesiology& Health Studies, Queen’s University, Kingston, K7L 3 N6 ON Canada; Department of Community Health and Epidemiology, Faculty of Health Sciences, Queen’s University, Kingston, K7L 3 N6 ON Canada

**Keywords:** Social network analysis, Maternal health, Reproductive health, Network mechanisms

## Abstract

Continuing high global maternal mortality and morbidity rates in developing countries have resulted in an increasing push to improve reproductive health services for women. Seeking innovative ways for assessing how positive health knowledge and behaviors spread to this vulnerable population has increased the use of social network theories and analysis in health promotion research. Despite the increased research on social networks and health, no overarching review on social networks and maternal health literature in developing countries has been conducted. This paper attempts to synthesize this literature by identifying both published and unpublished studies in major databases on social networks and maternal and child health. This review examined a range of study types for inclusion, including experimental and non-experimental study designs including randomized controlled trials, non-randomized controlled trials, quasi-experimental, cohort studies, case control studies, longitudinal studies, and cross-sectional observational studies. Only those that occurred in developing countries were included in the review. Eighteen eligible articles were identified; these were published between 1997 and 2012. The findings indicated that the most common social network mechanisms studied within the literature were social learning and social influence. The main outcomes studied were contraceptive use and fertility decisions. Findings suggest the need for continuing research on social networks and maternal health, particularly through the examination of the range of social mechanisms through which networks may influence health behaviors and knowledge, and the analysis of a larger variety of reproductive outcomes.

**Electronic supplementary material:**

The online version of this article (doi:10.1186/1742-4755-11-85) contains supplementary material, which is available to authorized users.

## Introduction

Death due to a pregnancy-related cause is 25 times more likely for women in developing countries than those in developed countries [1]. Inequalities in maternal mortality stem from economic, institutional, cultural and social circumstances, affecting the overall cost, access, and quality of healthcare services [2, 3]. In addition, women do not make reproductive health decisions in isolation, but are influenced by their broader networks of family, peer groups, and communities [4, 5]. Maternal and child health interventions are increasingly targeted to women in community and school settings yet evaluations of the efficacy of these health interventions efficacy have shown mixed success [6, 7]. Social network methods offer useful tools for analyzing the spread of health information and behaviors within health promotion programs in the developing world [8, 9].

Social networks refer to the patterns of relationships that exist among a set of actors. Social mechanisms play a key role in understanding the transmission, exchange, and circulation of goods within networks, whether these are physical, informational, or imitative [10]. Berkman and collegue’s conceptual model provides a useful framework for identifying the range of social mechanisms that may mediate the links between social networks and reproductive health. The model identifies four main mechanisms through which networks may impact personal health: *social support, social engagement, access to resources,* and *social influence*[11]. Although absent from Berkman et al.’s model, *social learning* might be considered an additional social mechanism through which networks can influence health.

Despite recognition of the importance of understanding the social channels through which health knowledge and behaviors spread, little research has examined social networks and the various social mechanisms by which reproductive health information and behavior may be transmitted. This study reviews the existing literature on social networks and reproductive health with the goal of assessing the potential added value in applying social network methods to the analysis of maternal and reproductive health outcomes in the developing world. Within this overall objective, the review aims to: (1) examine the research concerning social networks and reproductive health; (2) identify the specific social network mechanisms that researchers suggest influence behavioral or knowledge related outcomes in females of reproductive age; and (3) synthesize the results of those studies, identify gaps in research, and discuss their implications for future research and practice.

## Methods

This systematized review considered studies that involved the use of social network analysis to examine or improve the health of women of reproductive age in developing countries. Developing countries were defined according to United Nation’s Human Development Index (HDI) as those countries with a score below 0.79 on the HDI at the time that the research was published. Relevant social network literature for this paper studied women’s social networks as they impacted information or behavior in relation to reproductive health. Social network literature can be qualitative, e.g., relying on participatory interviews, or quantitative, e.g. using formal instruments such as name-generator surveys [12]. Both approaches were deemed acceptable for this review.

### Types of outcomes

Maternal or reproductive health was the outcome of interest. Under this umbrella term, outcomes might include birthing intentions, birth attendant decisions, family planning, usage of maternal and child health services, usage of referral systems within services, contraceptive knowledge and usage, child or adolescent pregnancy, and reproductive rights.

### Types of studies

This review considered all study types for inclusion such as experimental and non-experimental study designs including randomized controlled trials, non-randomized controlled trials, quasi-experimental, cohort studies, case control studies, longitudinal studies, and cross-sectional observational studies.

### Search strategy

**Figure 1:**
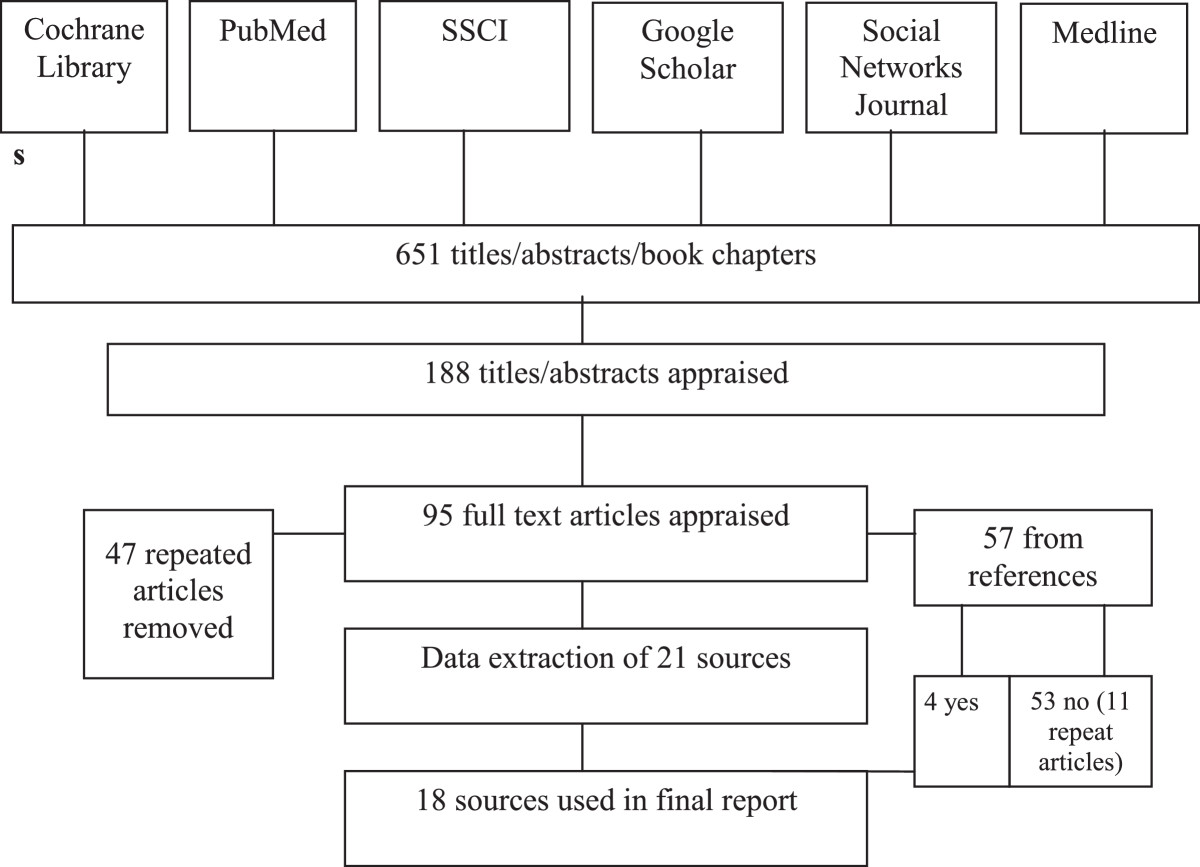
**Article search process flowchart.**

The search strategy aimed to identify both published and unpublished studies within major relevant databases for maternal and child health. No date parameters were placed on the search, ensuring that all potential articles were included to determine the depth of the research and its history. Boolean searches looking for the union of three sets of terms were conducted. These sets of terms were related to (1) the population (‘women’, ‘maternal’, ‘female health’, ‘fertility’), (2) the type of country in which they were conducted (‘developing’, ‘low-income’) or geographic region (‘Africa’, ‘Latin America and the Caribbean’, ‘Asia’, ‘Oceania’), and (3) social networks (‘social networks’; ‘network analysis’). The geographic groupings were based on those used by the United Nations Statistics Division (2013). A three-step search strategy was utilized in this review. An initial search of the databases of PUBMED, Medline, and Social Science Citation Index, the journal Social Networks, and the Cochrane Library was undertaken. Search terms were sought within the body of the article, abstract, and keywords. Secondly, references of each article found in the original search pool were examined to identify additional articles on maternal health and social networks. Third, Google Scholar was searched to identify any literature that may have been missed. Table 1 lists the key words used in the search; Figure 1 displays the search process used.

**Table 1:** **Search process documentation**

Data source	Documentation	Initial results	Chosen results
Scholars Portal Journals: *Social Networks*	Maternal health [anywhere]; Maternal health AND developing countries [anywhere]; Female health AND low-income [anywhere]; maternal [anywhere]; Maternal health AND Africa [anywhere]; Maternal health AND Latin America Caribbean [anywhere]; Maternal health AND Asia [anywhere]; Maternal health AND Oceania [anywhere];	3; 0; 0; 0; 6; 0; 0; 0; 0	0; 0; 0; 0; 0; 0; 0; 0
Cochrane Library (32 initial results; 10 chosen)	[Search Title, Abstract, Keyword] “social network” “maternal and child health”; [Search Title, Abstract, Keyword] “social networks” “maternal”; [Search Title, Abstract, Keyword] “social networks”; [Search Title, Abstract, Keyword] “maternal and child health” AND “network analysis”; [Search All Text] “maternal and child health” AND “network analysis”; [Search All Text] “social networks” “fertility”; [Search Title, Abstract, Keyword] “social networks” “maternal” “Africa”; [Search Title, Abstract, Keyword] “social networks” “maternal” “Latin America Caribbean”; [Search Title, Abstract, Keyword] “social networks” “maternal” “Asia”; [Search Title, Abstract, Keyword] “social networks” “maternal” “Oceania”	1; 1; 3; 0; 0; 0; 0; 0; 0; 0	0; 0; 0; 0; 0; 0; 0; 0; 0; 0
PubMed 9 repeat; 1 unable to access	“social network” AND “maternal and child health”[AND “developing”; ((“social networks”) AND (“fertility”)); ((“social networks”) AND (“maternal and child health”)); ((“social networks”) AND (“women”) AND (“developing countries”)); “social network*” AND “maternal health” AND Africa [All Fields]; “social network*” AND “maternal health” AND “Latin America Caribbean” [All Fields]; “social network*” AND “maternal health“ AND “Asia” [All Fields]; “social network*” AND “maternal health” AND “Oceania” [All Fields];	1; 43; 8; 38; 10; 0; 11; 3	0; 25; 1; 4; 6; 0; 4; 0
Medline (3 unable to access; 8 repeat)	(“social network*” and “maternal and child health”).mp. [mp = title, abstract, original title, name of substance word, subject heading word, keyword heading word, protocol supplementary concept, rare disease supplementary concept, unique identifier]; (“social network*” and “maternal and child health” and “developing countr*”).mp. [mp = title, abstract, original title, name of substance word, subject heading word, keyword heading word, protocol supplementary concept, rare disease supplementary concept, unique identifier]; (“maternal” and “social network*” and “low-income”).mp. [mp = title, abstract, original title, name of substance word, subject heading word, keyword heading word, protocol supplementary concept, rare disease supplementary concept, unique identifier]; (“social network*” and “fertility” and “developing countr*”).mp. [mp = title, abstract, original title, name of substance word, subject heading word, keyword heading word, protocol supplementary concept, rare disease supplementary concept, unique identifier]; (“social network*” and “fertility”).mp. [mp = title, abstract, original title, name of substance word, subject heading word, keyword heading word, protocol supplementary concept, rare disease supplementary concept, unique identifier]; “social network*” and “maternal health” and Africa).mp.; (“social network*” and “maternal health” and “latin america caribbean”).mp.; (“social network*” and “maternal health” and “Asia”).mp “social network*” and “maternal health” and “oceania”).mp.	8; 0; 10; 4; 32; 3; 0; 1; 0; 0	0; 0; 2; 2; 10 ; 0; 0; 1; 0; 0
Social Sciences Citation Index (SSCI) 2 unable to access; 2 repeats	Topic = (“social network”) AND Topic = (“maternal and child health”) Timespan = All Years. Databases = SCI-EXPANDED, SSCI, A&HCI, CPCI-S, CPCI-SSH, BKCI-S, BKCI-SSH.;; Topic = (“social network”) AND Topic = (“maternal health”) Timespan = All Years. Databases = SCI-EXPANDED, SSCI, A&HCI, CPCI-S, CPCI-SSH, BKCI-S, BKCI-SSH.; Topic = (“social network”) AND Topic = (“maternal and child health”) Timespan = All Years. Databases = SCI-EXPANDED, SSCI, A&HCI, CPCI-S, CPCI-SSH, BKCI-S, BKCI-SSH.;; Topic = (“social network”) AND Topic = (“fertility”) Timespan = All Years. Databases = SCI-EXPANDED, SSCI, A&HCI, CPCI-S, CPCI-SSH, BKCI-S, BKCI-SSH; Topic = (“developing countries”) AND Topic = (“female health”) AND Topic = (“network analysis”) Timespan = All Years. Databases = SCI-EXPANDED, SSCI, A&HCI, CPCI-S, CPCI-SSH, BKCI-S, BKCI-SSH; Topic = (social network*) AND Topic = (maternal health) AND Topic = (africa); Topic = (social network*) AND Topic = (maternal health) AND Topic = ( latin america caribbean); Topic = (social network*) AND Topic = (maternal health) AND Topic = (asia); Topic = (social network*) AND Topic = (maternal health) AND Topic = (Oceania)	3; 2; 2; 33; 8; 0; 3; 0	1; 2; 0; 17; 2; 0; 0; 0
Google Scholar (13 repeat)	“maternal and child health” AND “social network analysis”; “fertility” AND “social network analysis” AND “developing country”; “fertility” AND “female health” AND “social network” AND “low-income country”; “fertility” AND “female health” AND “social networks” AND “low-income country”; “maternal health” AND “social networks” AND “low-income country”; “maternal health” AND “social network analysis” AND “Africa”; “maternal health” AND “social network analysis” AND “Latin America” “Caribbean”; “maternal health” AND “social network analysis” AND “Asia”; “maternal health” “social network analysis” “Oceania”	137; 62; 3; 5; 51; 39; 11; 31	41; 10; 2; 2; 26; 15; 3; 8
African Index Medicus	“social network analysis”; “network analysis”; “social network”	0; 0; 0;	0; 0; 0
LILACS (7 repeated)	[Search Title, Abstract, Keyword] “social network” “maternal and child health”; [Search Title, Abstract, Keyword] “social networks” “maternal”; [Search Title, Abstract, Keyword] “maternal and child health” AND “network analysis”; [Search All Text] “maternal and child health” AND “network analysis”; [Search All Text] “social networks” “fertility”; [Search Title, Abstract, Keyword] “social networks” “maternal” “Africa”; [Search Title, Abstract, Keyword] “social networks” “maternal” “Latin America Caribbean”; [Search Title, Abstract, Keyword] “social networks” “maternal” “Asia”; [Search Title, Abstract, Keyword] “social networks” “maternal” “Oceania”	0; 58; 0; 19; 0; 0; 0; 0;	0; 4; 0; 0; 0; 0; 0; 0;
EMBASE (4 repeat; 2 unavailable)	(“social network*” and “maternal and child health”).mp. [mp = title, abstract, original title, name of substance word, subject heading word, keyword heading word, protocol supplementary concept, rare disease supplementary concept, unique identifier]; (“social network*” and “maternal and child health” and “developing countr*”).mp. [mp = title, abstract, original title, name of substance word, subject heading word, keyword heading word, protocol supplementary concept, rare disease supplementary concept, unique identifier]; (“maternal” and “social network*” and “low-income”).mp. [mp = title, abstract, original title, name of substance word, subject heading word, keyword heading word, protocol supplementary concept, rare disease supplementary concept, unique identifier]; (“social network*” and “fertility” and “developing countr*”).mp. [mp = title, abstract, original title, name of substance word, subject heading word, keyword heading word, protocol supplementary concept, rare disease supplementary concept, unique identifier]; (“social network*” and “fertility”).mp. [mp = title, abstract, original title, name of substance word, subject heading word, keyword heading word, protocol supplementary concept, rare disease supplementary concept, unique identifier]; “social network*” and “maternal health” and Africa).mp.; (“social network*” and “maternal health” and “latin america caribbean”).mp.; (“social network*” and “maternal health” and “Asia”).mp “social network*” and “maternal health” and “oceania”).mp.	13; 2; 11; 8; 76; 1; 0; 1; 0	2; 0; 0; 2; 0; 1; 0; 0; 0;

### Social mechanisms

Each article was examined to identify which social mechanism(s) were highlighted within the study. Berkman et al.’s model was used as the basis for identifying the different social mechanisms that may link social networks to health. These mechanisms were *social support, social engagement, access to resources,* and *social influence*[11]. S*ocial support* refers to the different types of support, whether informational, emotional, appraisal and instrumental, that may be available for individuals or groups as part of their social relationships. *Social engagement* pertains to levels of participation in meaningful social activities, either in leisure or productivity realms. *Access to resources* refers to network facilitation or restriction of access or opportunities of network members. *Social influence* is the social and institutional power within a society, whether overt or not, that exerts pressure over individual choice. In addition, social learning was included as a possible social mechanism. *Social learning* involves individual behavioural change mitigated by the exchange and evaluation of new information accrued through a person’s network. This, in turn, encourages the adoption of new innovations [13].

### Data extraction and synthesis

From the articles, the social network mechanisms, country location, study design, reproductive or maternal health outcome, and key findings were extracted from the articles.

## Results

The final pool of articles consisted of 18 papers published between 1997 and 2012. Table 2 provides a listing of the articles, the country location, sample size, social network collection methods, social network analysis methods, study design, variables, and identified mechanisms. Sample sizes among the studies ranged from 77 women to those with 10,003 individuals, including men and women. The majority of studies took place in Kenya (6) and Bangladesh (4). All studies were quantitative with the majority being cross-sectional and four longitudinal [14–17]. Concerning maternal health outcomes, 16 studies examined fertility choices, including the number of children a woman might have and their decisions about contraceptive use, while two examined the type of assistance obtained for childbirth [18] and women’s choice in place of delivery [19]. Studies focused primarily on the direct connection between a woman’s local social network and maternal health decisions [4, 13, 14, 16, 20–22]. However, four studies examined women’s fertility decisions and the influence of the social networks formed through migration [23], overall network density [24], interpersonal communication between spouses [25], and health campaigns targeting a whole population [17].

**Table 2:** **Key findings**

Author(s) (Date)	Context	Study design	Sample size	Social network collection methods	Social network analysis methods	Variables	Key Findings in Relation to Mechanisms	***p-*** value
Billari, Philipov & Testa (2009) [21]	Bulgaria	**Cross-sectional**	Men and women: 10,003, ages 18-34.	Name generator	Logistic regression models	Intentions to have a first and second child; attitudes, norms and perceived behavioral control related to fertility behavior.	Normative pressure, or the “perception of *social influence*” affected reproductive behavior, changing from being in favor of high childbirth rates to pro-contraception.	Norms; P = 0.00
Bove, Vala-Haynes, & Valeggia (2012) [5]	Mali	**Cross-sectional**	324 women, ages 15-80	Number of individuals a respondent identified.	Logistic and linear regression models	Pregnancy histories, women’s knowledge of contraception, and illness symptoms in the past three months and the treatment (if sought, financing and sources of social support).	*Social influence* in larger social networks resulted in increased pregnancy in the previous two years, associated with a larger social network.	Larger social network associated with increased odds of pregnancy during the previous 2 years, p < .01
Dynes, Stephenson, Rubardt & Bartel (2012) [4]	Ethiopia and Kenya	**Cross-sectional**	Ethiopia: 520 women; 300 men	Random generator.	Logistic regression model	Perceptions of current norms and community norms on current contraceptive use	*Perceptions of social norms* influenced reproductive behavior, including son preference and contraceptive use.	difference between women’s perception of the community ideal number of sons and their own actual number of sons is negatively associated with contraceptive use (Ethiopia OR 0.74, 95% CI 0.61–0.89; Kenya OR 0.77, 95% CI 0.66–0.89). *a higher score on the family planning perception of other’s approval index was significantly associated with current contraceptive use among men and women in Kenya (OR 2.03, 95% CI 1.35–3.05 and OR 1.36, 95% CI 1.06–1.74, respectively); this association, however, was not present among samples in Ethiopia.*
			Kenya: 655 women; 310 men					
Edmonds, Hruschka, Bernard, & Sibley (2012) [19]	Bangladesh	**Cross-sectional**	246 women, 18-49 years.	Network generator and network characteristics.	Logistic regression models	Place of delivery, whether home or facility	The collective advice of others, or *social influence,* whether correctly perceived or not, affected birth decisions of women.	Skilled Birth Attendant Endorsement by network *p* = .000
Gayen & Raeside (2007) [18]	Bangladesh	**Cross-sectional**	694 women who had at least one child,	Name generator.	Logistic regression models	Experience of neonatal death and choice of assistance for delivery	*Social influence* impacts choice in type of assistance while giving birth.	Degree centrality in relation to unqualified assistance *P* = 0.00; degree centrality in relation to professional assistance *p* = .01.
Gayen & Raeside (2010) [20]	Bangladesh	**Cross-sectional**	694 women currently married of reproductive age	Name generator.	Logistic regression models	Current use of contraception	Both *social learning* and *social influence* impacted family planning decisions.	Network members’ approval of contraception, *p* < 0.05. Network members’ encouragement to use contraception, *p* < 0.05. Discussion frequency on contraception with network members, *p* < 0.05.
Kincaid (2000) [14]	Bangladesh	**Longitudinal**	860 married women, age 14-49.	Random generator.	Logistic regression model; Conditional (static-score) multiple regression analysis	Modern contraceptive use	A social network approach, specifically group discussions in key opinion leader’s homes, allowed for increased *social influence* to accelerate rate of change concerning contraceptive use.	Social network approach change in ideation *p* < 0.001, change in contraceptive use, *p* < 0.001.
Kohler, Behrman & Watkins (2001) [24]	Kenya	**Longitudinal**	694 women currently married	Name generator	Logistic regression; Measures of network density.	Family planning use	More heterogeneous groups with high amounts of activity were dominated by *social learning* and more homogenous groups are dominated by *social influence.*	In low-density Owich, Kawadhgone and Wakula South, the % users influence on family planning is *p* < 0.01; In high-density Obisa, density influences family planning *p* < 0.01.
Lindstrom & Munoz-Franco (2005) [23]	Guatemala	**Cross-sectional**	2871 women, age 18-35.	Random generator.	Multilevel logistic regression model	Contraceptive knowledge	*Social learning* is integral in areas where networks increased in heterogeneity. Key actors also influenced this learning.	Migration experience, family migration networks, and community urban out-migrant networks were statistically significant at precdicting the number of modern contraceptive methods known, *p* < 0.05.
Madhavan, Adams & Simon (2003) [13]	Mali	**Cross-sectional**	502 women, aged 15-45,	Random generator	Ordinary least-squares regression; logistic regression	Two fertility-related outcomes – completed fertility and contraceptive use	Homogenous networks facilitated *social influence* as a mechanism for diffusion; ‘gatekeepers’ generally dictated these societal norms and had more influence than others.	Ever use of contraceptives contraceptives: Presence of mother *P* < 0.05; % of network who are natal kin, *p* < 0.05; $ of network who are conjugal kin *p* < 0.01; % of network who live outside villag, *p* < 0.001.
Musalia (2003) [25]	Kenya	**Cross-sectional**	200 to 323 women, younger than 50	Name generator.	Logistic regression analysis.	Educational heterogeneity; membership in voluntary organization; network size; contraception use.	*Social influence* of kin groups affected spousal discussion of contraceptive use, but as gave way to social learning as new ideas were embraced.	Being a member of a social group: Kakamega, p < 0.05; Murang’a, p < 0.01.
Musalia (2005) [26]	Kenya	**Cross-sectional**	557 women and 536 men	Name generator.	Logistic regression analysis	Ever use of contraception and current use of contraception.	*Social influence* both hindered and helped the adoption of reproductive behaviors	Current use of contracetion, ntowrk advices use of family planning, p < 0.01; ever use of contraception, network advices use of family planning, p < 0.01.
Sandberg (2005) [29]	Nepal	**Cross-sectional**	77 currently married women, younger than 50	Name generator	Logistic-regression	Desiring more children.	*Social learning* and *collective social experiences* influenced actor decisions and behaviors.	Desiring more children impacted by network infant mortality, p < 0.05; and any child died in last birth interval, p < 0.01.
Valente, Watkins, Jato, Van Der Straten, & Tsitsol (1997) [27]	Cameroon	**Cross-sectional**	495 women, under the age of 45	Name generator.	Use logit-regression models.	Whether respondent ever-used a contraceptive, a clinic-based method, and a non-clinic based method.	*Social influence* and *social learning* were important within networks, though *influence* is heightened within associations due to encouragement between members.	Perceived approval of contraction, have used contraception, have encouraged network partners to use all *p* < 0.0001.
Behrman, Kohler & Watkins (2002) [16]	Kenya	**Longitudinal**	497 women; 324 men	Name generator.	Logit model	Whether a respondent was currently using contraception (at the time of the survey).	*Social learning* was the primary means of transmitting information through a network.	At least one family planning uer in the network *p* < 0.05; Number of remaining family planning users in the network, *p* < 0.01.
Boulay & Valente (1999) [22]	Kenya	**Cross-sectional**	2,217 women, aged 15-49; 2,152 men, aged 15-54	Random generator.	Logistic Regression models	Family planning knowledge, attitudes, and practices.	Extended social networks led to high amounts of transmission of *family planning information passed through community groups.*	Family planning knowledge, approval, use and discussion among members of clubs: know 5 modern methods, p < 0.001, and talked about family planning with anyone p < 0.01, with core network only, p < 0.05, and with core and extended networks, p < 0.001.
Valente & Saba (1998) [17]	Bolivia	**Longitudinal**	First sample: 2300 youngest men and women present in household; Second sample:800 residents in Potosi.	Name generator.	Regression model with demographic controls.	Family planning awareness; reproductive health knowledge; reproductive health attitudes; family planning intention; interpersonal communication; current use of contraceptives.	*Social learning* in the form of mass media campaigns were associated with behavior change for individuals who have networks with low amounts of contraceptive use.	Network exposure and current use of contraception (p < 0.01) was associated with family planning awareness p < 0.01, reproductive health knowledge p < 0.01, reproductive health attitude p < 0.01, family planning intention p < 0.01,
Godley (2001) [28]	Thailand	**Cross-Sectional**	1,563 women aged 18-35 who had been married 10 years or less.	Random generator.	Logistic regression models; multilevel networks	Choice in contraceptive.	The specific social network of extended kin influenced contraceptive choice both through both *social learning* and *social influence*.	Method choice without television with p < 0.05, and method choice with television, p < 0.05.

### Synthesis of findings

Due to the non-homogenous nature of the studies themselves, a meta-analysis could not be performed. Instead, the following section provides the principle findings and an overview of the research organized according to the main mechanisms identified. Using Berkman’s model, 50% of the articles were classified as examining *social influence*, 28% *social learning*, and 22% examined a combination of both. *Social support, social engagement,* and *access to resources* mechanisms were generally absent.

### Social influence

Social influence, like other mechanisms, can potentially hinder or help the adoption of positive reproductive behaviors [26]. Social influence may operate more powerfully within more homogeneous, dense, closed networks [13]. Such networks can be characterized by closely tied groups or ‘cliques’ in which new ideas and behaviours may spread rapidly and influence network members that play an important role in shaping cultural norms [13]. Within the literature, it was suggested that cliques provide boundaries within a social group that may protect members from deleterious external influences through social influence of non-adoption [14]. Cliques may also utilize the mechanism of social influence to limit access to new innovations, even if beneficial, derived from outside contacts [14]. An additional article suggested that the same influence keeping members from adopting health behaviors and knowledge from outside their homogenous group can constrain or pressure women’s choices in relation to their reproductive health to align with other beliefs, practices or values of their social network [13, 21]. Other literature additionally supported the suggestion that social influence impacts reproductive health behaviors. One study showed that the collective advice of others within a social network, whether correct or not, affected birth decisions of women [19]. This was bolstered by an additional study, which showed that social influence specifically affected women members of associations within larger non-kin-based networks, increasing their likelihood to use similar methods as their network counterparts [27]. However, another study within a different context showed that while a group was homogenous, this did not influence knowledge of contraception itself for network members [4, 5].

Density and the type of network also was shown to affect both social influence and social learning [20]. Village kinship networks were shown to create the overall cultural context within which behavioral choices were made through social learning. However, household kinship networks supposedly either facilitated or hindered access to information and resources related to reproductive health through social influence [28].

### Social learning

Social learning is often considered one of the main mechanisms by which new and innovative information is transmitted through social networks [17]. There are a number of studies that highlighted social learning as a mechanism for information or behavior change. One study found that infant mortality rates influences family planning decisions through social learning – information gained about high mortality rates through social networks, typically through member’s experiences, leads to an increased production of children and greater family size [29].

Research has also suggested that the younger a woman is, the more likely she is to be influenced through social learning [13, 22]. The networks themselves were also shown to be heterogenous [13, 22]. However, an additional study found that the mechanism of social learning in relation to community network members does not influence individual network members as strongly as when information is provided by members in the household, specifically in relation to child-rearing practices [25]. This study’s finding was bolstered by an additional piece of literature which found that social learning was stronger in kin-based social networks [13].

As stated in the previous section, density and type of network is suggested to affect the degree of social learning within a network. It was also suggested that villages with higher levels of economic diversity and greater heterogeneity are characterized by a greater degree of social learning. Here, economic diversity along with the greater in-and out-migration of individuals may act to introduce villagers to a range of ideas, opinions, and choices regarding maternal health and fertility. Therefore, information and behaviors that come from outside a network may diffuse more broadly within local networks that are more heterogeneous [23, 24].

## Discussion

This review revealed the paucity and fragmentation of reproductive health and social network literature currently available. In relation to the objectives of the review, it was determined that current literature is non-homogenous and occurs in a variety of different areas of research and varying geographic locations. All studies used a quantitative methodology and primarily examined fertility choices within the area of reproductive health outcomes. Identification of specific social network mechanisms influencing health related outcomes within the studies showed only two were researched – social learning and social influence. Other mechanisms, such as *social engagement, social support* and *access to resources* were absent from the literature. The following paragraphs address the third objective of this study, identifying gaps in research and discussing the findings implications for practice.

### Strengths and weaknesses of the study

There are several strengths of this review. First, the comprehensiveness of the literature search ensures that the key, relevant English-language research was found. Second, the literature itself encompasses a wide range of academic fields, not only remaining only within social network analysis or health promotion. Third, this review forms a basis for researchers and health workers to continue studying and utilizing social network analysis in their research and practice. The study aids in highlighting the gaps in how researchers have studied the mechanisms linking social networks to reproductive health and provides a guide to how research can move forward to provide a greater understanding of the relationship between the two. Fourth, this review relies on a widely accepted theoretical framework within the social network analysis field for assessing mechanisms linking networks to health. Fifth, this compilation of literature is unique. As such, however, there are no other relevant systematic reviews or meta-analyses from which to compare the strengths and weaknesses of this study, as it is the first summarizing the literature concerning social networks and reproductive health.

There are several limitations of this study to note. First, due to diverse disciplines and differing terminology throughout the literature, some articles may have been missed in the search process. Although reproductive health and maternal health were used as search terms within the title, abstract and body of the paper, the search may have missed articles focusing on specific outcomes, such as usage of services, access to care, or contraceptive use. Second, grey literature, reports, and unpublished studies were not included in this review. Third, although not a limitation of the search itself, there may be a publication bias in the research on social networks and reproductive health in that negative or null results may not be published. If negative or null results were more likely when researchers examined network mechanisms other than social influence or learning, it may have resulted in the overrepresentation of such studies in the literature. Fourth, all studies included were quantitative in nature. However, the authors did not limit the review to only quantitative research. The authors recognize that qualitative research would bolster available literature, giving it more depth and understanding in relation to complex social ties [30]. Fifth, there was a lack of interventions within the literature, with most research being observational in design, rather than involving or evaluation the implementation of network interventions to improve reproductive health.

### Meaning of the study

#### Using networks to guide interventions

Our synthesis suggests that social learning and social influence mechanisms may play different roles depending on the network environment. For example, in heterogeneous, open social networks, research has suggested that social learning tends to be a more salient mechanism whereas in homogenous, dense networks, social influence may play a greater role. To be most effective, health interventions should seek to align specific strategies with the network mechanisms presumably at play in particular environments. In a network where social influence may be the main mechanism for spreading information and behavioral norms, interventions that target influential kin members of an individual’s network may be an effective means to increase knowledge and modify reproductive health behaviors, based on the literature in this review. However, to leverage social learning mechanisms, a health promotion and intervention method that targets the general public may be the best means of diffusing innovations and new behaviours. For example, in fragmented network contexts with little contraceptive use, a mass media campaign may be the best means of reaching the greatest amount of individuals [17].

### Examining multiple contexts

Two studies stood out from the rest due to their multilevel analysis of social forces dictating women’s reproductive health. These studies examined how different network contexts, e.g. home or community, affected reproductive behavior [23, 28]. Such findings have implications for the design of more ecological-level interventions and development strategies aiming to improve the reproductive health of women. For example, community networks may be targeted, with key members potentially used as family planners and ‘influencers’ of the health behaviours of a local network.

### Incorporating geographical barriers

Physical geographical barriers also impact maternal health care by limiting access to health care or information on currently recommended practices, hindering a social network approach to the delivery of health knowledge and behaviors. The equitable delivery of services between urban and rural areas is often difficult due to the poor infrastructure in rural areas [31]. Combining a geospatial approach with social network analysis when examining reproductive health would allow researchers to examine the spatial and social diffusion of information, which has not occurred within the research up to date.

### Researching men’s social networks

This usage of specific network contexts and significant individuals within them also has gendered implications. Few studies have examined both men and women in tandem. Yet, men can have a significant impact on reproductive decisions and often hold considerable decision-making power in the household concerning contraception. Future research should examine the importance of men’s social networks when it comes to the social norms surrounding men’s beliefs and practices on contraceptive use and reproductive health.

### Unanswered questions and future research

Research on social networks and maternal or reproductive was examined to identify which social mechanisms – *social support, social engagement, access to resources, social influence* and *social learning —* were used to explain the links between networks and maternal health. This review showed that the two most prominent mechanisms in maternal health and social networks literature were *social learning* and *social influence*. However, this review also revealed a variety of challenges currently and for future research in the field.

To date, research on social networks and health suggests a range of possible mechanisms linking networks to health outcomes. Nevertheless, the current state of social network and reproductive health literature is not large and tends to be restricted to the study of social influence and learning mechanisms. The investigation of additional mechanisms would add depth and breadth to the understanding of the relationship between social networks and reproductive health behaviors and knowledge in developing countries.

The means of measuring social networks in relation to reproductive health is an additional area that is a current challenge. Most studies utilized a name generator as a means of gathering network information, relying on ego-centric methods. Full network methods and snowball methods were generally not utilized within the literature. Full network methods is challenging to undertake, especially in developing nations. Utilizing a variety of collection methods would add breadth to the field.

Additionally, concerning methodology, current literature primarily examined one level of a network, without gathering multi-modal information, such as how an individual works within a structure, such as organization or society. Examining networks on macro, meso, and micro levels of analysis allows for a multifaceted analysis simultaneously. This is a challenge to research, as it requires greater resources and, again, can be difficult to perform in developing countries.

Homophily, or the tendency of similar people to associate with others like themselves, was also found to be a challenge for this body of literature when distinguishing between social influence or social selection. The principle of homophily creates challenges for current and future research since it is difficult to measure whether social networks are creating certain reproductive behaviors that align with the norms and values of a culture, or whether individuals have chosen to associate with others who have similar health behaviors and beliefs as themselves.

Specifically within this body of literature, the disparate contexts of each study is a challenge. Each cultural context may influence women differently and, therefore, generalizing findings to the overall body may not be applicable to certain networks in different countries.

## Conclusions

It is only within the past two decades that health research has examined the link between reproductive knowledge and behavior and social networks. At present, research has focused primarily on contraceptive use and family planning outcomes. Social influence and social learning tend to be the main mechanisms that researchers currently use to explain the importance of social networks for reproductive health. Future research on networks and maternal reproduction would benefit from a more comprehensive treatment of the mechanisms hypothesized to link social networks to health. A more comprehensive understanding of the social network influences on reproductive and maternal health would facilitate the design, implementation and evaluation of maternal health interventions in the developing world.

## Authors’ original submitted files for images

Below are the links to the authors’ original submitted files for images. Authors’ original file for figure 1

## Abbreviations

HDI: Human development index.
