# Supplementary figures and images for: Social networks and female reproductive choices in the developing world: a systematized review
Source: Reprod Health. 2014 Dec 10;11:85. doi: 10.1186/1742-4755-11-85 (PMC4275947; doi:10.1186/1742-4755-11-85)

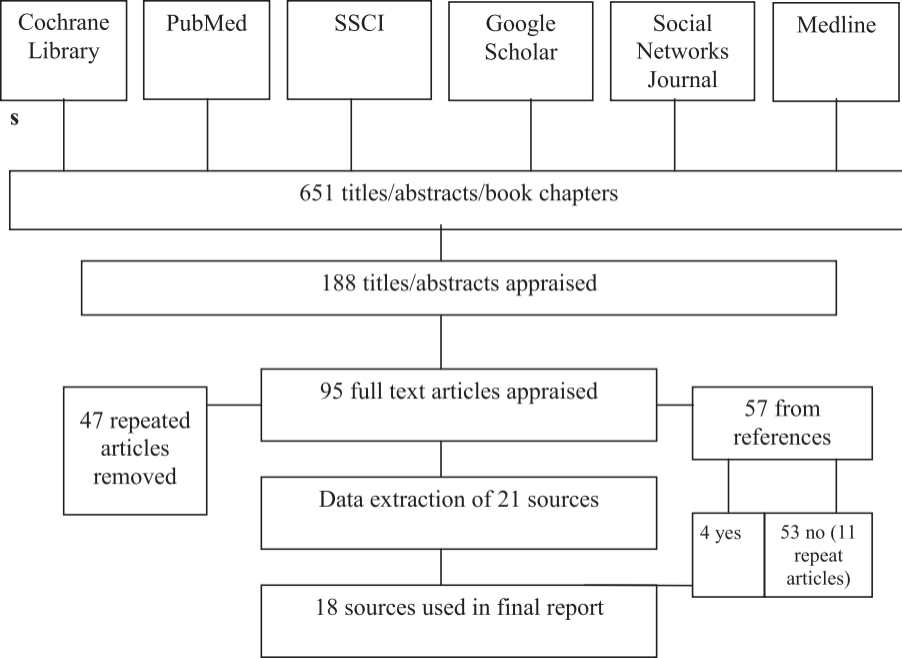

Supplement: Supplementary file 1 — Authors’ original file for figure 1 [file 12978_2014_334_MOESM1_ESM.pdf]
